# The effect of subsidized malaria treatment among under-five children in the Buea Health District, Cameroon

**DOI:** 10.11604/pamj.2019.33.152.16832

**Published:** 2019-06-27

**Authors:** Hedwig Eposi Nkwenti, Marcelin Ngowe Ngowe, Pius Fokam, Joseph Nkfusai Fonyuy, Sylvester Ndeso Atanga, Ngwayu Claude Nkfusai, Brenda Mbouamba Yankam, Joyce Mahlako Tsoka-Gwegweni, Samuel Nambile Cumber

**Affiliations:** 1 Department of Public Health and Hygiene, Faculty of Health Sciences, University of Buea, Buea, Cameroon; 2 Department of Microbiology and Parasitology, Faculty of Science, University of Buea, Buea, Cameroon; 3 School of Nursing & Public Health, College of Health Sciences, University of KwaZulu-Natal, Durban, South Africa; 4 Faculty of Health Sciences, University of the Free State, Bloemfontein, South Africa; 5 Section for Epidemiology and Social Medicine, Department of Public Health, Institute of Medicine (EPSO), The Sahlgrenska Academy at University of Gothenburg, Gothenburg, Sweden; 6 School of Health Systems and Public Health, Faculty of Health Sciences, University of Pretoria Private Bag X323, Gezina, Pretoria, 0001, South Africa

**Keywords:** Health care services, subsidized malaria treatment, under-five children, user fee

## Abstract

**Introduction:**

Access to free diagnoses and treatments has been shown to be a major determinant in malaria control. The Cameroon government launched in 2011 and 2014 the exemption of the under-fives' simple and severe malaria treatment policy to increase access to health care and reduce inequality, so as to reduce the mortality related to malaria among the under-fives. This study assessed the effect of providing free malaria treatment in the Buea health district.

**Methods:**

This retrospective and cross sectional study was carried out in the Buea health district. Aggregated monthly data from (2008-2010) before and (2012-2014) after the implementation of free malaria treatment was compared, to assess the attributable outcomes of free treatment. A semi-structure questionnaire was also used to assess barriers faced in providing free malaria treatment services by health care workers. Data was collected using a semi-structure questionnaire and a data review summary sheet. The data was analysed using Epi-Info 7, Excel and SPSS (Statistical Package for the Social Sciences) version 20.0 for Windows. All statistical tests were performed at 95% confidence interval (significance level of 0.05).

**Results:**

Increase utilisation of health care; as general and malaria related consultations (by 5.7% (p=0.001) witnessed an increase after the implementation of free malaria treatment services. Severe malaria hospitalisation also increased, indicating that most caregivers used the health facility when complications had already set in, which could have led to no significant reduction in mortality due to malaria among under-five children (4.4%, p=0.533).

**Conclusion:**

Utilisation of health care increased; as consultation and morbidity rate increased after the implementation of free malaria treatment services. Communication strategy should therefore be strengthened so as to better disseminate information, so as to enhance the effectiveness of the program. There is the need to make a large-scale study to assess the impact of subsidized malaria treatment.

## Introduction

Malaria infection is a public health problem and a major cause of death in sub-Sahara Africa [1-3]. Most countries in Africa are faced with the challenge of early diagnosis and treatment of malaria before complications set in [4-6]. Malaria infection is the leading cause of morbidity and mortality in children, due to the high prevalence within this age group [1, 7-10]. Over 1,200 under-five children die every day from malaria, with an equivalent of 50 children in an hour and 80% of death from 16 countries in Africa and since 2000 there has been a decline in 15 countries [6, 11]. The under-five mortality due to malaria between 2000 and 2015, fell by 65% globally, which is an estimation of 5.^9^ million children saved [6]. An estimated 6.2 million malaria deaths have been averted globally since 2001 [4, 12]. The burden of malaria in most countries has greatly reduced with scaling up of prevention, diagnosis, and treatment procedures [13]. Much has been done by Africans to scale up prevention and closing the gaps such as: increased in funding for malaria control, procurement and distribution of effective means for prevention and treatment which are associated with falls in malaria burden. Also, the change from a failing drug (chloroquine) to a more effective drug (sulphadoxine plus pyrimethamine or an artemisinin combination) led to immediate improvements; in others, malaria reduction seemed to be associated with the scale-up of insecticide-treated bed nets and indoor residual spraying [13]. Malaria is endemic in Cameroon, where it is the leading cause of morbidity (41%) and mortality (43%) [14]. According to the National Malaria Control Programme (NMCP), malaria accounts for 50%-56% of morbidity and 40% of deaths among children less than five years of age, constituting a heavy burden on the health care system and economy. In 2012, the prevalence of malaria in Cameroon was 33.3% while mortality and morbidity were set at 30%. It also accounts for 45% of under-five mortality [14].

The Cameroon government launched the policy of free malaria treatment which was adopted in Cameroon in 2011 and 2014, for the treatment of simple and severe malaria respectively and in other countries as well [15-17]. The free malaria treatment, is a package that includes subsidized diagnosis of malaria and treatment for under-five children and pregnant women for severe and simple malaria provided in all health facilities [13, 15, 16, 18]. Free treatment policy is beginning to be the option for improving access to health care for most diseases affecting the most vulnerable groups in sub-Sahara Africa [19-22]. Many African countries made use of user fees in 1980s and its negative effects on access to health care services is well documented such as low utilisation of health care services and increase mortality [16, 20]. Due to the negative effect user fee had on the quality of care receive, there is a recent shift in health financing debates in Africa [22-25]. Prompt diagnosis and adequate treatment is essential to reduce morbidity and mortality related to malaria among vulnerable groups as stipulated by WHO [26], since mothers, could identify signs and symptoms of malaria (fever) in children and take them to the nearest health facility within 24 hours of unset of symptoms [7, 25]. The introduction of this policy is similar to what was adopted in other countries in Asia and other parts of Africa to combat the high prevalence, mortality due to malaria and to increase accessibility of health care especially in under-five children and pregnant women [26-31]. After the introduction of the policy in Cameroon, an evaluation was carried out in the Adamawa region from 2011 to 2012 and result show an increase in utilisation from 19.6% to 32.9% though some barriers were also identified with regards to staffs' attitudes [16, 20, 32, 33].

## Methods

**Study area:** Buea is located in the South West Region of Cameroon and it is the capital of the region, it is one of the 18 districts in the South west region. It is situated on the eastern slopes of Mount Cameroon with a total surface area of 870 square kilometres. Buea has an equatorial climate with two season: the dry and wet season. Its population was about 169,745 inhabitants in 2017, with under-five population of approximately 23002 (13.6%) [34]. It is a cosmopolitan town with other ethnic groups but mainly inhabited by the Bakwerians who have lived around the slopes of Mount Cameroon for many years.

**Study design and setting:** this was a retrospective and cross-sectional study, in which health facilities monthly records were reviewed to assess the effect of the implementation of free malaria treatment on under-fives health care utilisation and mortality due to malaria. It was done by reviewing registers within a period of 3 years before exemption of user fee and comparing them to 3 years after exemption of user fee, which are the periods within 2008-20^10^ and 2012-2014 respectively.

**Study duration:** this study was carried out from March to August 2017, which is within six months.

**Sample size determination:** the study included reviewing existing data in selected health facilities and collecting data on cases that came in with fever and malaria test was ran on them.

**Sample size for data review:** no sample size was calculated for data review, rather health facilities monthly report (all aggregated reports) from 2008-2010 and 2012-2014 within the selected clusters were reviewed. Out of 31 health facilities in the Buea health district, 26 within the selected clusters were used. Questionnaires were also administered to health facilities.

**Inclusion criteria:** all health facilities within the selected clusters.

**Exclusion criteria:** health facilities not within the selected clusters. Facilities with incomplete records.

**Sampling technique:** a multistage sampling method was used, whereby each health area served as a cluster. Five clusters were selected by simple random sampling and they included: Buea Town, Buea Road, Molyko, Tole, and Muea health areas. From each clusters all facilities that met the selection criteria were used for the reviewed.

**Data collection and processing:** semi-structured questionnaire and a data summary form were used to collect data used for this study.

**Review of records:** data review summary form was used to get information which was keyed into an excel tally sheet on the situation of free malaria services within the time frame chosen and its effect on mortality, hospitalisation due to severe malaria and morbidity rate.

**Administration questionnaires:** questionnaires were administered among consented participants who agreed to participate in the study. The questionnaires captured data on socio-demographic characteristics, barriers perceived in providing free malaria treatment services by health care providers.

**Data management and statistical analysis:** a semi-structured questionnaire and a data summary form was used to collect data. Data was entered in Excel and EPI info version 7.0 and analysis was done using Excel and SPSS (Statistical Package for the Social Sciences) version 2.0. Information from data review was keyed into an excel tally sheet and analysed using excel. It was later on imported into excel for analysis in SPSS aversion 20.0 for Windows for statistical analysis. The Z-test for comparing groups proportion, chi-square test and fisher test were used to compare the differences between categorical variables. All statistical tests were performed at 95% confidence interval (significance level of 0.05). Data was presented in the form of tables and charts.

**Ethical considerations:** ethical clearance was obtained from the Institutional Review Board of the Faculty of Health Sciences of the University of Buea. Administrative authorization was also obtained from the South West Regional Delegation of the Public Health and the District Health Services of Buea. The purpose of the study and the role of the participants were well explained on the consent form to the participants and participation only took place after the participant read and signed the informed consent forms voluntarily. Participating in this study did not expose the participants to any major risk. They benefited from knowledge on malaria prevention and management. Confidentiality was ensured by using codes to identify study participants rather than their names.

## Results

**Effect of free malaria treatment in improving under-five malaria related outcomes:** generally, utilisation of health care services by under-five increased after the implementation of free malaria treatment services. As general consultation rose from 26393 to 31157 after the implementation of free malaria treatment service among under-fives. Likewise, malaria related morbidity among under-fives increased from 43.3% (before) to 49.0% (after) implementation free malaria treatment services (p<0.001) (Table 1).

**Table 1:** Comparisons before and after the implementation of free malaria treatment among under-five children

	BEFORE (2009-2010)			AFTER (2012-2014)			Statistic	
Element	N	n	%	N	n	%	Z- Test	P-value
Under-five malaria related morbidity rate	26393	11436	43.3	31157	15263	49.0	13.561	0.001
Simple malaria consultation rate	26393	9564	36.2	31157	10605	34.0	5.511	0.001
Severe malaria related consultation	26393	1763	6.7	31157	4658	14.9	31.4	0.001
Hospitalization rate for severe malaria	3808	1416	37.2	6055	3570	59.1	87.847	0.001
Under-five malaria related mortality rate	66	21	32.0	113	31	27.4	0.623	0.533

(In the table above N and n refer to the denominator and the numerator of the indicator respectively. The rate is the ratio of n to N expressed in percentages)

**Figure 1:**
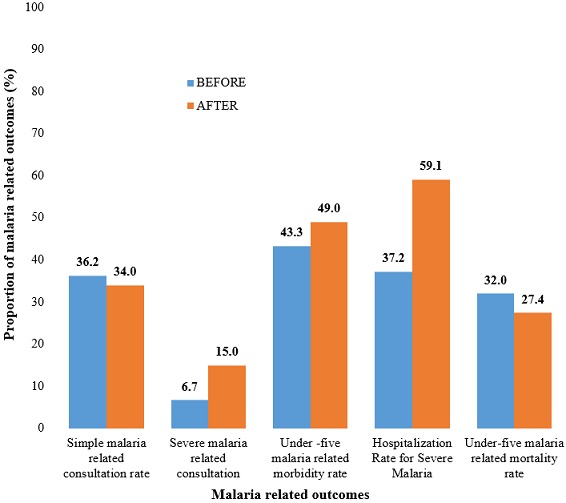
Comparison before and after the implementation of free malaria treatment among under-five children

**Malaria related outcomes among the under-fives before and after the implementation of free malaria treatment services:** stratifying morbidity rate by the type of malaria infection indicated a significant increase in the number of severe malaria related consultation from 6.7% to 14.9% (p<0.001). Meanwhile, severe malaria hospitalisation rate increased from 37.2% to 59.1% before and after respectively. However, malaria related mortality rate dropped slightly from 32% to 27.4% (p=0.533) (Figure 1).

**Barriers in providing free malaria treatment by health facilities:** a total of 11 health facilities were involved in the study, there were 5(45.5%) public health facilities as well as 5(45.5%) private facilities and 1(9.1%) faith based health facilities. Most of the questionnaires were answered by chief of centres 3(27.3%) as well as nurses 3(27.3%). It was closely followed by medical doctors 2(18.2%) and general supervisor 1(9.1%). Majority 8(72.7%) had correct knowledge about free malaria treatment policy while 2(18.2%) did not know about free malaria treatment. Majority of the health facilities 10(90.9%) received free malaria treatment as compared to 1(9.1%) which did not receive. Most of the health facilities 9(81.8%) reported treating simple malaria for free while 1(9.1%) did not treat for free. Strategy of informing the care-givers involved informing during prescription 4(36.4%), informing them when purchasing drugs 2(18.2%) followed by passing the information during home visit, use of posters and flyers with a proportion of 1(9.1%) each. Lack of commodities was a mild constrain among the health facilities [6(60.0%)]. Clients behaviour was serious in private health facilities [3(75.0%)] as compared to public health [0(0.0%)] facilities though it was not significant (p=0.158). Majority [5(50.0%)] of the health facilities were mildly faced by adherence to treatment. Cost sharing was rated at seriously, severe and mild with 1(10.0%), 0(0.0%) and 9(90.0%) respectively. Lack of funds was an issue with most health facilities (severe [3(30.0%)] and serious [3(30.0%)]), it was more serious among private health facilities [2(40.0%)] as compared to public health facilities [1(25%)] though it was not significant.

## Discussion

Exemption of fee for malaria treatment among the under-fives in Buea health district resulted in an increase service utilization for curative care as general and malaria related consultations by under-fives increased after introduction of free treatment services [35]. This is as a result of improved treatment seeking behaviour which promoted early diagnosis. This is similar to the result of the study carried out in Sudan where health services utilisation increased at each level of user fee exemption. Also, in Kangaba, Mali, user-fee exemption for under-five irrespective of the illness led to increase service utilisation [36]. This findings also ties with the result of the study carried out in Cameroon (Adamawa) [16]. Increased service utilisation led to an increase in the malaria related morbidity rate among under-five by 5.7%. This could be as a result of the fact that, the free malaria policy has it that any child presenting with fever should be systematically tested for malaria and has made provision for faster and better testing options such as the use of a rapid diagnostic test (RDT) which could be done by an unskilled staff especially in facilities which lack staffs. In so doing the number of test carried out increased coupled with adequate record keeping [15]. Stratifying the morbidity rate based on simple and severe malaria, it was realised that, the number of simple malaria was quite reducing after the implementation, meanwhile severe malaria significantly increased. This is as a result of the fact that, most caregivers delayed in seeking adequate health care, they brought their children for consultation when complications had already set in [37] or because malaria is a well-known disease most care-givers knew treatment options, so they started treatment at home but provided low dosage or incomplete treatment which cause the infection to become chronic [38]. Also, the nature of their jobs as most participants were self-employed, they will only go to the health facility when the child’s situation became serious [38]. This conforms to results from [39], where occupational activities was strongly associated to delay in seeking care [39].

Hospitalisation of under-fives due to severe malaria significantly increase after the implementation of the free malaria treatment policy, which was as a result of the increased severe malaria consultation. An indication that most care-givers did not come early to the health facility following the onset of fever either due to the fact that they did not know about free malaria treatment services as indicated in the household interview or prefer trying home treatment especially when fever was not serious [39]. Most of the participants also complained of long waiting time which could be another barrier in accessing prompt health care when child had fever. This is similar to findings in a study carried out in Ghana whereby there was no difference in the anaemic level between the intervention and control group [40]. This is an indication that the provision of free malaria treatment had no effect on reducing complications of malaria infection. There was no significant reduction in mortality among under-fives after the implementation of the free malaria treatment policy. This outcome is similar to the findings reported by [40], where there was a slightly higher mortality in the intervention area as compared to the control area [41]. This defers with results from the study carried out by Ponsar F et al. [36], whereby increase utilisation of health services let to a decreased reduction in mortality among under-five [36]. Meanwhile, study carried out in Ghana indicated no effect on mortality outcome [40]. This insignificant decrease can be explain by under-reporting of death resulting from malaria and poor record keeping before the implementation of the free treatment policy in the study area as most health facility could not provide register as far back as 2012 [41]. It could also be as a result of increase severe malaria as reported by the study.

Lack of commodities was not a limiting factor in receiving treatment contrary to what was reported in Kenya [20]. Poor communication and transfer of information from health personnel to care-givers due to the fact that strategies employed to inform people may not be actively used. these strategies could easily be forgotten at the time of intervention [39], this therefore support the needs for increase sensitisation. Though some health facilities faced challenges in providing free treatment, these challenges were not significant. This may be due to the fact that most health facilities did not return their questionnaires but lack of drugs and RDT were quite higher among public health facilities since this were the most visited, since they offer subsidised health care services. Lack of funds was quite an issue since some public institution revealed that selling drugs brought in funds to cater for the facility needs. Malaria being the leading cause of morbidity in Cameroon and Buea in particular so much funds were being lost by the facility when providing free treatment. A study carried out by Ridde V et al. [40], reveals that most health facilities started reinstating user fee so as to raise funds for the health facility [41].

**Some limitations in our study include:** our study was limited to the Buea Health District, and we recommend that an extensive study should be made so as to come out with more significant findings on the effect of the policy.

## Conclusion

In this study we assessed the outcomes of subsidized malaria treatment among the under-fives by comparing the outcomes three years before and three years after the rollout of the free malaria treatment policy. The study also administered a semi-structure questionnaire to assess the knowledge of care-givers and barriers to free malaria treatment. Findings of the study indicated increase utilisation of health care as general and malaria related consultations witnessed an increase after the implementation of free malaria treatment services. This was accounted for by improved and increased testing, better reporting strategies which accompanied the free malaria treatment package. Most care givers brought their children to the health facility when complication had already set in, as severe malaria consultation rate and severe malaria hospitalisation increased after the implementation of free malaria treatment services. Most care-givers did not know about the policy (65.1%), majority of those who knew about the policy complained that, free malaria treatment services were accompanied by long waiting time and poor health workers attitude. There was therefore a need to improve information dissemination on the free malaria treatment services

### What is known about this topic

- After the introduction of the policy in Cameroon, an evaluation was carried out in Adamawa region from 2011 to 2012.
- Rwanda also offer free malaria treatment due to a rise of the number of cases from 800,000 in 2012 to 3.9million in 2015/2016.
- In an experimental study carried out in Sudan 2004, to assess the effect of the different levels of user fee exemptions at 25%, 50% and 75% on health service utilisation and treatment seeking behaviour for pregnant women and children under-five in Sinnar State of Sudan

### What this study adds

- Malaria in under-five children has contributed greatly to increase morbidity and mortality due to the high cost required for treatment especially among this age group.
- The study compared treatment outcome of malaria resulting from subsidised treatment in the under-fives.
- Assessment of knowledge about free malaria treatment among care-givers and challenges faced in receiving and providing subsidised services.
